# Resolving the paradox of unipolar induction: new experimental evidence on the influence of the test circuit

**DOI:** 10.1038/s41598-022-21155-x

**Published:** 2022-10-06

**Authors:** Christof Baumgärtel, Simon Maher

**Affiliations:** Department of Electrical Engineering and Electronics, University of Liverpool, Liverpool, UK

**Keywords:** Physics, Engineering, Electrical and electronic engineering

## Abstract

A novel experiment has been devised shedding new light on the phenomenon of unipolar induction, also known as “Faraday’s Paradox”. This is a topic which continues to fascinate scientists and engineers with much debate continuing to this day. In particular, the question of the field co-rotating with the magnet or remaining stationary remains unsettled and supporting evidence exists for both positions. In this study, we present a novel experimental apparatus that includes, for the first time, the relative motion of the measurement circuit including the closing wires, as well as the magnet and disc respectively. The results show that the closing wire needs to be considered as part of the problem, which enables the apparent paradox associated with this phenomenon to be resolved. However, it remains impossible to tell if the field co-rotates with the magnet or if it remains stationary. Instead, direct electron interaction is considered as a viable alternative to resolve remaining paradoxes.

## Introduction

The year 2021 marked the 200th anniversary of the discovery of electromagnetic rotation^1^ first reported by Michael Faraday in 1821^2^ when he partially immersed a permanent magnet in mercury and connected a battery to the assembly. This remarkable discovery paved the way for what would become two of the most important applications in human history: the electric motor and generator. While modern designs of these applications consist of rotor and stator configurations with several magnetic poles, Faraday would later continue to investigate a generator utilising only one of the magnet’s poles and a conducting disc rotating around its cylindrical axis: the unipolar induction machine, also known as the Faraday generator. Even to this day, roughly 190 years after Faraday first reported a voltage induced across a disc that is spinning in a magnetic field^3^, unipolar induction is still being discussed and debated among scientists and engineers. As this phenomenon is synonymously titled “Faraday’s paradox”, it indicates the confusion it has caused and consequently sparked debates about the fundamental nature of the problem with different opinions about the underlying physical cause. In this regard, physicists are especially divided about the question as to whether the magnetic field co-rotates with the magnet or if it remains stationary upon the magnet’s rotation.

**Figure 1:**
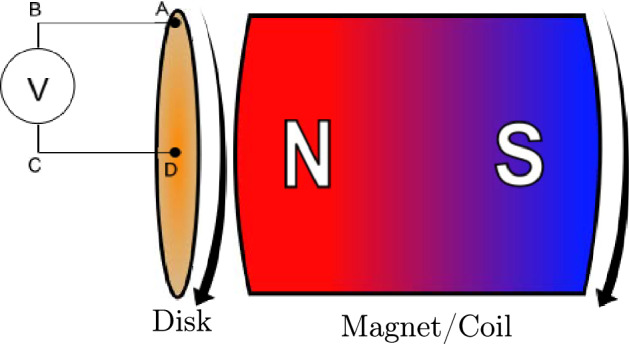
Sketch of a unipolar induction machine (also known as a Faraday generator).

To recall the phenomenon and associated paradox, an illustration is given in Fig. 1 where a magnet is situated next to a conducting disc, both of which are free to spin around their cylindrical axis. Usually, the following three cases are considered for discussion:

(i) The disc is rotating and the magnet remains stationary. A voltage can be measured across the radius of the disc.
(ii) The disc remains stationary and the magnet rotates. No voltage can be measured across the disc.
(iii) Both disc and magnet rotate at the same speed and direction, and again a voltage is observed across the disc.

Faraday originally considered that the field remains stationary when the magnet rotates and the disc cutting the field lines causes an Electromotive Force (EMF) to appear which can be observed as the induced voltage across the disc. Later he changed his point of view, thinking that the field lines co-rotate with the magnet instead. Especially when one considers rectilinear motion of a magnet, it appears that the field will move with the magnet, therefore expecting it to co-rotate around the cylindrical axis seems like a logical deduction. However, for this case, a voltage is expected to be induced for case (ii), but not for case (iii) and thus an apparent paradox is established. If the field lines are assumed to remain stationary upon the magnet’s rotation instead, the disc would cut the field lines in cases (i) and (iii), where indeed a voltage is found to be induced, but this also contradicts the observation of rectilinear motion where the field will follow the magnet’s movement. The phenomenon is also often discussed as paradoxical because it is said to violate Faraday’s flux rule, as it shows an induced voltage where none is expected. This is exemplified by Feynman who labelled unipolar induction as an exception to the flux rule^4^.

There are also other paradoxes associated with the flux rule, e.g. Hering’s paradox^5,6^ and Cullwick’s experiment^7,8^, but it has been discussed in^9^ how the application of the correct mathematics can avoid the creation of any apparent paradoxes of induction experiments in the first place. Even though certain phenomena are often labelled as special cases or exceptions to specifically the flux rule, it was shown that they are only labelled as such due to omissions and incomplete application of the necessary mathematics or portions of the circuit. In particular the omission of certain parts of the circuit is further investigated experimentally in this article and the results can support the notion that there is no such paradox when these are regarded as part of the problem. Furthermore, it is interesting to note that particularly Faraday’s paradox seems to be linked to the field being rotationally symmetric, and the magnet rotating around the symmetry axis. If, for example, one considers a horseshoe magnet instead, and spins its poles in succession, the field must clearly move with the poles, similar to rectilinear motion of the magnet. Another example would be the rotation of a quadrupole magnet, as investigated by Leus and Taylor^10^ where they concluded that the field rotates with the magnet. Thus, an apparent paradox only occurs when a magnet rotates around the field’s axis of symmetry. However, there is a more fundamental underlying question to this problem, namely if the field is a real physical entity or not? A discussion about the field moving or rotating with the magnet can only be had if the field is physically tangible. If instead it is only a mathematical tool and not real, then consequentially it cannot move with the magnet and thus any discussion regarding its movement is meaningless.

Hence, the present paper is seeking to investigate the phenomenon and the possibility of the field co-rotating with the magnet. As such we have developed a novel experimental setup involving a specifically designed detector circuit that can be rotated in the magnetic field. Similarly, the disc and magnet can be rotated in this new Faraday generator assembly so that the influence of each component on the induction can be tested in all of the conceivable configurations.

## Brief review of theoretical and experimental viewpoints

There are diverging views about this phenomenon in the literature, both in theory and in practice. Ultimately the question of the field’s rotation (or not) with the magnet can only be answered experimentally, if it can be answered at all. Several experimental investigations about the phenomenon exist^3,10–31^ and an extensive list of references giving a historical overview, including theoretical considerations and experimental results, can be found in a review by McDonald^9^. Some more recent investigations^10,28^ have supported the field co-rotating with a magnet, whereas other results^29–31^ found agreement with the field remaining stationary.

Kelly^28^ performed experiments where a return wire connecting to the measurement device would be routed parallel to the disc and tested different configurations where the return wire is arranged in a “zig-zag” pattern. It was claimed that the routing affected the results in such a way that it could only be explained by the field co-rotating with the magnet as the return wire was being cut by the field lines at multiple sections. However, these findings have been challenged by Macleod^29^, who reported that Kelly’s results could not be reproduced and the routing of the wires had no influence on the observed voltage (albeit it was neither clear how nor which parts of Kelly’s work specifically were recreated). Generally, Macleod used a twin-disc setup with ring magnets attached to each disc in opposing polarity and measured the induced voltages for individual and twin-disc rotations. It was concluded that these measurements agree with the field remaining stationary.

Leus and Taylor^10^ utilised a ring magnet and an annular iron yoke with an air gap which were both rotated. A voltmeter was connected to a stationary probe through the air gap and onto a copper cylinder sitting inside, but isolated from, the ring magnet. The other contact of the voltmeter was connected to a brass ring below the magnet, and the brass ring was in turn connected to the copper cylinder. They further tested the rotation of a quadrupole ring magnet with a current loop. The conclusion from the resulting data was that the field is spinning with the magnet.

Müller^30^ has performed experiments that included an assembly of conducting magnets in oscillatory motion, moving back and forth on a circular arc instead of full rotation of the setup. An external portion of the circuit parallel to the magnets was connected to an amplifier, and the magnets were situated in a yoke that effectively shielded the external circuit path from the magnetic field. It was concluded that the results support the field remaining stationary.

Chen et al.^31^ came to the same conclusion following an investigation with a twin-disc setup where each disc was sitting next to a ring magnet rotated in opposite directions. The discs could rotate independently with the magnets remaining stationary or together with the magnets. Measuring the voltages induced in the system, they concluded that their results agreed with the field remaining stationary upon rotation of the magnet.

As can be deduced from this selection of experimental results in the academic literature there seems to be evidence to support both hypotheses and no clear conclusion can be drawn. This only serves to intensify the paradox. Theoretically this problem has been analysed from different viewpoints: the field regarded as stationary^4,32–41^ or moving^10,28,42–45^; Faraday’s law not being applicable^4,22,46^ as well as the contrary^36,47,48^; discussions as to whether special relativity^8,26,27,49,50^ must be used or not^17^; the application of general relativity^36,51^; and quantum mechanical explanations^52–54^. For a further discussion of explanations for unipolar induction based on SRT, a review by Bordoni^55^ is recommended. Special focus is given to the Wilson experiments^56,57^ on induction in a rotating dielectric in a magnetic field, and the Einstein–Laub paper^58^. On this matter the views of Laue, Föppl and Becker are summarised, which are in support of a special relativistic explanation of unipolar induction.

Similarly, there are several mathematical analyses that show how the correct expression and value of induced EMF can be obtained in more than one way^9,10,59–61^. There is also an approach by Montgomery^62^ based on the charge carriers and energy conservation that can predict the EMF, and the problem has further been analysed with a direct-action approach based on Weber electrodynamics^63–67^. Weber himself derived Faraday’s law from his force formula^63–65^ as was acknowledged by Maxwell in his *Treatise*^68^ [article 856, p. 486] and Wesley has shown how Weber’s force can predict unipolar induction in general^66^. More recently, Weber’s force has been successfully applied specifically to a spinning disc and stationary electromagnet setup^67^.

So while there are a plethora of approaches and mathematical descriptions that correctly predict the EMF observed, it does not yet alleviate the confusion about the physical cause of the phenomenon. One approach to explain the fundamental underlying cause is based on the work of Assis and Thober^69^ who make the claim that the entire circuit, including the measurement device and closing wires, need to be included in the analysis to properly explain unipolar induction. That is, in Fig. 1, the path ABCDA needs to be considered. It is then relative motion between the circuit portions BC (including closing wire and measurement device) and part DA (the disc) that determines the appearance of an EMF. They analysed the problem theoretically using Weber electrodynamics and correctly predicted the usual cases (i), (ii) and (iii). This approach is promising as it is based on a physical aspect of the experiment that is often overlooked, thus the entire experimental arrangement is considered. Following this promising line of enquiry, further analysis of the problem including a field perspective, is considered in the next section.

## Theoretical analysis: influence of the closing wire

In our analysis we acknowledge the closing wire, respectively the measurement circuit, as part of the problem. Allowing each of the elements, disc (DA), magnet and closing wire (BC) to rotate, leads to 8 possible configurations that can be analysed from the perspectives of moving field (MF), stationary field (SF) and direct-action (Weber electrodynamics). The results of this analysis are summarised in Table 1, where $\omega$ indicates the rotational motion of a component and 1 (i.e., true) indicates an expected EMF. Each possible case can now be considered in turn:

**Table 1:** Predicted EMF in 8 possible cases for three different theoretical interpretations.

Case	DA	BC	Magnet	SF	MF	Weber
1	0	0	0	0	0	0
2	$\omega$	0	0	1	1	1
3	$\omega$	$\omega$	0	0	0	0
4	$\omega$	0	$\omega$	1	1	1
5	0	$\omega$	0	1	1	1
6	0	$\omega$	$\omega$	1	1	1
7	0	0	$\omega$	0	0	0
8	$\omega$	$\omega$	$\omega$	0	0	0

BC and DA relate to the parts of the measurement circuit that can move, where BC is the closing wire and DA the disc, as illustrated in Fig. 1.

**Case 1:** None of the parts move, therefore no voltage can be induced.

**Case 2:** Only the disc is rotating. For both stationary and co-rotating field perspectives the disc cuts the field lines once, the disc gets polarised due to the magnetic force, whereas the closing wire stays neutral. Thus, a current can flow and an induced voltage can be observed. In Weber’s theory, the charges in the disc are moving and consequentially see an EMF from the magnet which polarises the disc while the closing wire stays neutral and a current can flow, so a voltage is observed.

**Case 3:** Disc and measurement circuit are rotating. The field lines are being cut twice in this case, once by the disc and once by the closing wires of the measurement device. This means that both parts of the circuit are polarised in the same way as they both see a magnetic force, however, current cannot flow in this case as the polarisation is in the same sense, so the circuit is never completed and the voltage cannot manifest. Similarly in a direct-action approach, both parts of the circuit are polarised in the same way and current cannot flow.

**Case 4:** Disc and magnet are rotating. Considering the field remaining stationary, the disc is cutting the field lines and in turn polarised while the closing wire remains neutral. So a current flows and the voltage is induced across the disc. If the field is regarded as co-rotating with the magnet, the field lines now cut the closing wire and polarise it while the disc remains neutral. But again, a current can flow and a voltage is induced. With Weber’s force, the charges moving with the disc see an EMF due to the microscopic circulating charges (i.e., Ampèrian currents) in the magnet, which polarises the disk while the closing wire stays neutral, thus a current can flow and the voltage appears.

**Case 5:** Only the measurement circuit (and its associated closing wire) is moving. Similar to case 2, the field lines are cut only once, this time by the closing wire, which gets polarised while the disc remains neutral, a current can flow and a voltage is induced. With a direct force approach, the force is acting on the moving charges in the closing wire and causing it to polarise. As the disc remains neutral, a current can flow and a voltage is observable.

**Case 6:** Measurement device and magnet are rotating. If the field remains stationary, then the closing wire is cutting the field lines, polarises with the disc remaining neutral and a voltage is induced. Vice versa, should the field be moving with the magnet, the field lines cut the disc, polarise it and induce a voltage. With Weber’s theory, it is the charges in the closing wire that experience a force due to their movement and the presence of the charges within the permanent magnet. The closing wire will then be polarised while the disc remains neutral, a current can flow and a voltage is induced.

**Case 7:** Only the magnet rotates. For the field remaining stationary this is the same as case 1. For the field co-rotating this is similar to case 3 where the field lines cut the circuit twice and no voltage appears. Considering the Weber force, disc and closing wire will be polarised the same way, a current cannot flow and a voltage cannot manifest.

**Case 8:** For each of the approaches, there is no relative motion and thus no EMF is expected.

As can be seen with this analysis, the three different theories actually predict the same results, but the physical reasons for an appearing EMF are different. This is especially apparent when case 4 and 6 are compared, both field approaches predict the appearance of an EMF but for opposite physical reasons. However, with this analysis the three approaches agree that a voltage can be observed when the disc (DA) and closing wire (BC) are polarised differently.

To the knowledge of the authors, no scientific publication has investigated the influence of a rotating closing wire and the measurement circuit to a sufficient degree. The closest experiments known are those of Kelly^28^, Müller^30^ and Valone^70^. While Kelly’s results were contended by Macleod^29^, neither of the two specifically tested the rotation of the measurement device itself. In Müller’s tests the external portion of the circuit was effectively shielded from the magnetic field and the measurement device itself remained stationary. Valone has reported an experiment with a one-piece Faraday generator where an indicator LED was connected to the conducting magnets. Keeping the LED detector circuit stationary detected a voltage, whereas when the detector was spinning together with the magnets no voltage could be detected, which tests (and is in compliance with) cases 4 and 8 only. To this end, we have devised a new experimental arrangement to test whether the rotation of the closing wire and measurement circuit have any influence on unipolar induction in a typical Faraday generator configuration consisting of magnets, disc and measurement device.

## Apparatus

**Figure 2:**
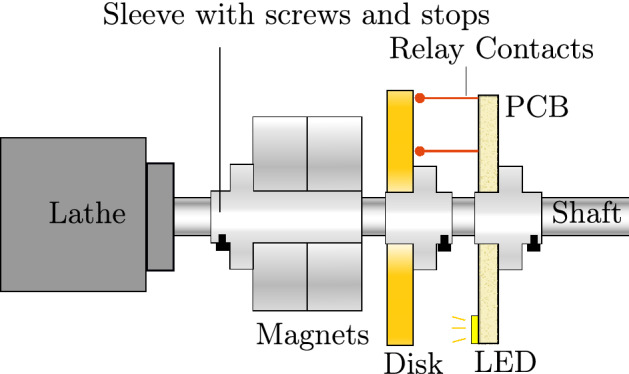
Sketch of the experimental setup.

**Figure 3:**
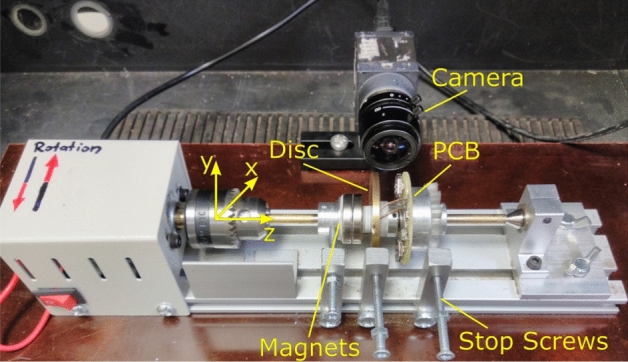
Photograph of the experimental setup. A right-handed system of coordinates is placed so that the brass shaft is coaxial with the z-axis. Neodymium magnets and brass disc are each glued to an aluminium sleeve, the measurement PCB is screwed to an aluminium sleeve with nylon screws. Each of the sleeves sits on bearings and can rotate with the shaft or be fixed with the help of dedicated stop screws as indicated in the photograph.

The experimental setup is sketched in Fig. 2 and a photograph depicted in Fig. 3. Its core component is a mini lathe with a brass shaft on which two magnets, a brass disc and a measurement PCB sit. Each of these three components is fixed to an individual sleeve each running on a bearing, so that a part can be held stationary by an external stop screw or rotate with the shaft by use of two grub screws fixing it to the shaft. This allows for each part to be fixed or rotate independently to test the eight cases described in the previous section.

The magnets used were two N42 neodymium magnets, (outer diameter 25 mm, inner diameter 16 mm, thickness 5 mm, part number F2516-1) with a remanence $B_r$ $=$ 0.4 T acquired from Bunting eMagnets (Hertfordshire, UK). The disc is made of brass, with 5 mm thickness and outer diameter of 42 mm, while the PCB is slightly larger at 43 mm outer diameter and employs an amplifier circuit to light an LED that acts as a visual indicator for when a voltage is induced in the circuit. Soldered onto the PCB are relay contacts with a brass tip that are bent into a Z-shape to spring load the contacts to ensure continuous contact to the disc separated by approximately 10 mm along the radius. It is noted that the magnetic field for this setup is not perfectly homogeneous due to the distance between magnets and circuit, and the ferromagnetic nature of the bearings used. However, this does not affect the rotational symmetry of the field, which is the stronger requirement for unipolar induction.

A dedicated circuit has been designed to qualitatively test the existence of an EMF in this setup. On the circular PCB the relay contacts are soldered onto 3 mm wide copper tracks that form the closing wire and run along the radius until they branch off into the inputs of an LTC2053 In-Amp that amplifies the input voltage induced in circuit ABCDA. The output of the amplifier is connected to a yellow high efficiency LED (ROHM SML-D11YWT86, 1.85 V, 2 mA) with a current limiting resistor. Power is supplied to the amplifier through two button batteries BR1225. A schematic of the circuit and the layout of the PCB can be found in the supporting information, Fig. S1 and S2 respectively.

A preliminary test was carried out with the disc spinning in the magnetic field to determine the necessary amplifier gain. With a Rhode & Schwarz HMC8012 digital multimeter a maximum potential difference of about 2 mV was measured across the entire available 14 mm radial distance on the backside of the disc where the relay contacts connect. In practice the relay contacts are separated by around 10 mm and thus the measurable voltage is closer to about 1.5 mV. Based on these values the amplifier gain is set at 2000 to ensure operation of the LED. The contacts are subject to some slight positional inaccuracies due to them being soldered onto the copper tracks and experiencing centrifugal forces in the cases where the PCB is rotating, but with the choice of gain a sufficient margin was ensured so that the LED could turn on should the induced voltage be reduced slightly due to small shifts in position.

As a consequence, due to a gain of 2000 on the amplifier, the relay contacts then act like antennae and the LED can light up very weakly in its base state due to surrounding noise in the lab environment. However, when the contacts are shorted (as they are when they are touching the brass disc) the LED turns off and an expected “0” base state of the setup can be observed. Additionally, a low pass filter was installed as part of the circuit to attenuate 3 dB at 620 Hz but had no significant effect and no observable noise reduction. As long as the contacts were properly pushed against the disc with the help of their spring-loaded nature, a constant connection to the disc could be readily achieved upon rotation, with the LED turned off as per the base state.

The lathe has seven different speed settings determined by the supply voltage ($V_S$), of which the no load configuration has been measured to determine the rate of rotation. The speed was measured with a digital stroboscope (Model Lutron DT-2239A, Lutron Instruments, Taiwan). The measured speeds are listed in Table 2.

**Table 2:** Measured rotational speeds of the lathe in no load condition.

$V_S$ in V	$RPM$	$\omega$ in $\hbox {rad s}^{-1}$
12	4717±25	493.9±2.6
15	5847 ± 37	612.3 ± 4.0
16	6228 ± 28	652.2 ± 3.0
18	6988 ± 33	731.8 ± 3.5
19	7326 ± 32	767.2 ± 3.4
20	7723 ± 15	808.8 ± 1.6
24	9240 ± 10	967.6 ± 1.0

Several control tests have been performed to ensure the working condition of the apparatus. As the lathe is driven by a DC motor, its rotational direction can be readily reversed by switching the polarity of the supply. Thus, it was determined how the rotational direction polarises the disc with the chosen magnets in the setup. For all experiments the orientation of the magnets remained fixed, so that the polarity of the EMF could be controlled through the rotational direction. With the coordinate system indicated in Fig. 3, the lathe can be spun in clockwise (CW) or counterclockwise (CCW) direction. It was found that for CCW rotation the disc polarises with the negative charges on the circumference and the positive charges in the centre. As expected, the opposite occurs when rotated CW, the negative charges are found in the centre and the perimeter of the disc is charged positively. This was determined with the help of a HMC8012 multimeter by observing the sign of the detected voltage.

**Figure 4:**
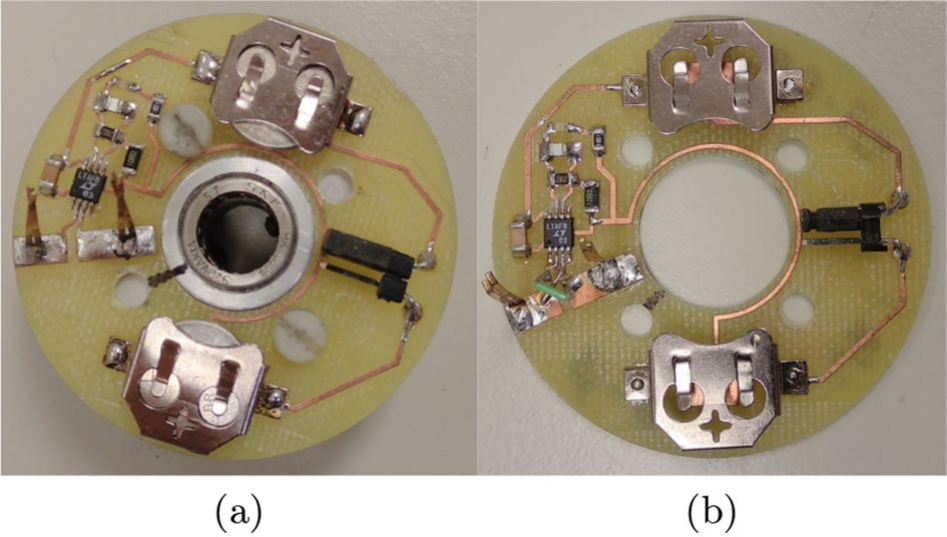
Photograph of the circuit board with LED and amplifier circuit (**a**) normal polarity (NP), (**b**) inverted polarity (IP).

Following this, the polarity of the In-Amp was chosen for the detector circuit. It was set so that the outer contact is connected to the inverting input of the amp and the centre contact to the non-inverting input, further called “Normal Polarity” (NP). A second configuration was chosen where the outer contact connects to the non-inverting input and the central contact connects to the inverting input, called “Inverted Polarity” (IP). Thus, two PCBs have been manufactured to account for NP and IP, photographs of these are shown in Fig. 4.

The functioning of the NP circuit was tested with an external voltage source. The negative terminal was connected to the outer contact (inverting input) and the positive terminal to the central contact (non-inverting input) to simulate a disc that is polarised negatively on the perimeter and positively in the centre. As the cables connected to the contacts increased the noise picked up, the LED increased in brightness as a consequence, but it could still be observed that when switching the voltage source on and supplying 2 mV, the brightness increased yet again. Next, a negative voltage (−2 mV) was supplied and this led to the LED switching off completely, which was expected, as the amp was designed with the negative side of the batteries as a 0 (zero) V reference and cannot output negative voltages. This confirms the correct operation of the circuit.

Next, the disc and PCB were rotated in absence of the magnet. First the disc spinning alone was tested while the PCB was kept stationary, and after the disc remained fixed and the PCB was rotated. This was tested for different rotational speeds, both CW and CCW directions and both amplifier polarities, the LED remained switched off without a magnet present.

## Results

With this, the eight configurations as given in Table 1 were tested, first CW and CCW with NP amplifier setting. The results are shown in Table 3 and in case 2 and 4 where the disc was spinning CCW a voltage could be observed as expected. At first it was surprising that in cases 5 and 6, where the measurement circuit itself is rotating, no voltage could be detected for CCW rotation in NP, even though it was expected. However, a voltage could be detected in cases 5 and 6 but only for CW rotation, whereas all other cases could not be detected due to the NP setting of the In-Amp preventing a voltage output. Before explaining how and why these results depend on the polarity of the amplifier, we shall present the results of the tests made with the IP amplifier setting, as this shows the reproducibility and acts as a control for the previous results. With IP we could again check CW and CCW directions. In this case it was CW rotation where a voltage, due to the disc spinning, was observed and CCW rotation where a voltage, due to the measurement PCB rotating, was observed - exactly opposite to the NP results. These results are summarised for both polarities and rotational directions in Table 3.

**Table 3:** Results for testing cases 1 to 8 in both rotational directions with both polarities of the amplifier.

Case	DA	BC	Magnet	Observed State of LED			
				CCW Rotation		CW Rotation	
				NP	IP	NP	IP
1	0	0	0	0	0	0	0
2	$\omega$	0	0	1	0	0	1
3	$\omega$	$\omega$	0	0	0	0	0
4	$\omega$	0	$\omega$	1	0	0	1
5	0	$\omega$	0	0	1	1	0
6	0	$\omega$	$\omega$	0	1	1	0
7	0	0	$\omega$	0	0	0	0
8	$\omega$	$\omega$	$\omega$	0	0	0	0

An $\omega$ indicates movement of the respective part and a 1 (true) represents an observed “on” state of the indicator LED, meaning a voltage is observed.

**Figure 5:**
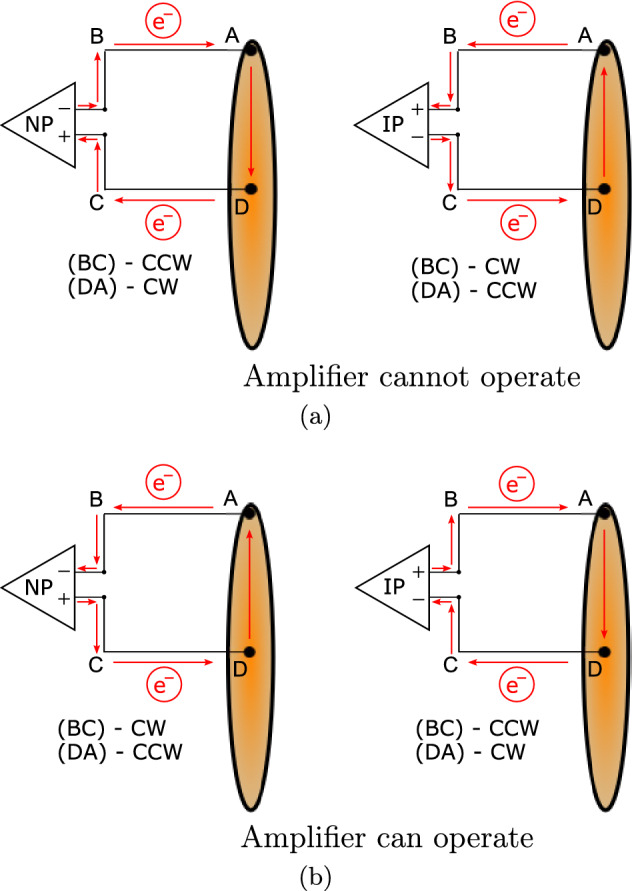
The electron flow in circuit ABCDA determines the operation of the amplifier, which in turn depends on the polarisation of the disc (DA) or closing wire (BC) due to the rotational direction in the magnetic field: (**a**) states in which the amplifiers cannot operate and no voltage can be observed; (**b**) states the amplifiers can operate in and a voltage can be observed.

To explain why case 5 and 6 in NP show an observable voltage only when the rotational direction is inverted, one needs to consider the flow of electrons in the circuit ABCDA. Intuitively, we might expect that the disc and closing wire will polarise in the same way for a given field and rotational direction due to the magnetic force. This first assumption led the authors to believe that a voltage would be observable for CCW rotation and NP of the amplifier. However, it is precisely because the closing wire is polarised in the same sense as the disc that the LED does not turn on in this case. Considering the electron flow, when the measurement PCB is spinning in CCW direction with NP amplifier setting, the electrons in the closing wire flow to the perimeter of the copper track, away from the inverting input, through the disc and the central contact and then again outwards on the copper track towards the non-inverting input (see Fig. 5a). This means the electrons are flowing the wrong way, away from the input they should flow into, so the LED cannot turn on. If, however, the rotational direction is reversed (CW) and the electrons now flow towards the centre in the closing wire, it will turn on, because they are flowing away from the non-inverting input, through the central contact, back through the disc and then from the periphery of the closing wire inwards to the inverting input (Fig. 5b), enabling the LED to turn on. This leads to the conclusion that the closing wire does indeed polarise as expected in a magnetic field and leads to different operational states of the amplifiers, as shown in Fig. 5.

Two additional tests have been performed to check the induced voltages in cases 5 and 6: (i) increasing the field strength by adding additional magnets to the setup, as it was considered that perhaps the field was too weak at the position of the closing wire due to it being further away from the magnets than the disc; and (ii) measuring the intensity of the LED for case 5.

Two additional N42 neodymium magnets with larger radii (OD 40 mm, ID 25 mm) and of similar field strength were added to the setup and set closely behind the closing wire, at approximately the same distance that the disc sat from the regular magnets. Spinning the NP amp in CCW direction with the additional magnets in the same polarity as the regular ones showed that the LED still remained off for both case 5 and case 6, despite the now significantly increased field strength at the closing wire. When turning the extra magnets to the opposite polarity of the regular ones the LED still did not turn on as the EMF was likely too weak due to one field interfering with the other. Next, spinning the NP amp in CW direction where the LED turns on in case 5 and 6 was tested with the additional magnets. In both case 5 and 6 having the extra magnets, the same polarity as the regular ones, increased the brightness of the LED. Vice versa, having the extra magnets face the opposite way decreased the brightness as one field counteracted the EMF of the other.

**Figure 6:**
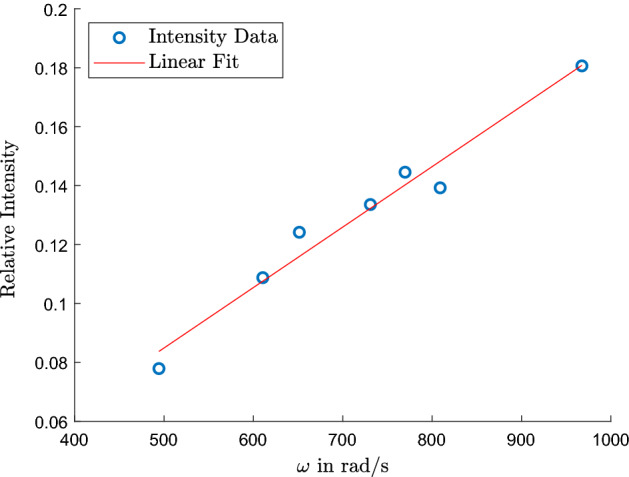
Relative intensity of the LED in case 5 for different speeds of rotation. An intensity value of 1 represents an entirely white picture.

To further confirm the induced voltage in the closing wire by the regular magnets only, intensity measurements in an enclosure have been made for case 5 at different rotational speeds with a Thorlabs scientific camera D1024G13M. An image of the spinning PCB was captured for each speed setting on the lathe to analyse the brightness of the trace the LED leaves on the camera image. The mean intensity of the greyscale values was calculated in *MATLAB* (MathWorks, MA, USA) and scaled against a value of 255, which would represent a fully white picture. The relative intensity values can be seen in Fig. 6 and a linear dependence on the rotational speed is clearly visible from the data, as is expected. The linear fit was found to be

$$\begin{aligned} I_{rel} = \omega \cdot 2.05 \times 10^{-4} - 0.0177 \end{aligned}$$

with an $R^2$ of 0.967. Furthermore, it was also observed that the signal decreased when the magnet was moved away from the disc slightly, which was especially noticeable at higher rotational speeds. This dependence confirms that there is indeed an EMF induced in the closing wire by the magnets.

## Discussion

**Figure 7:**
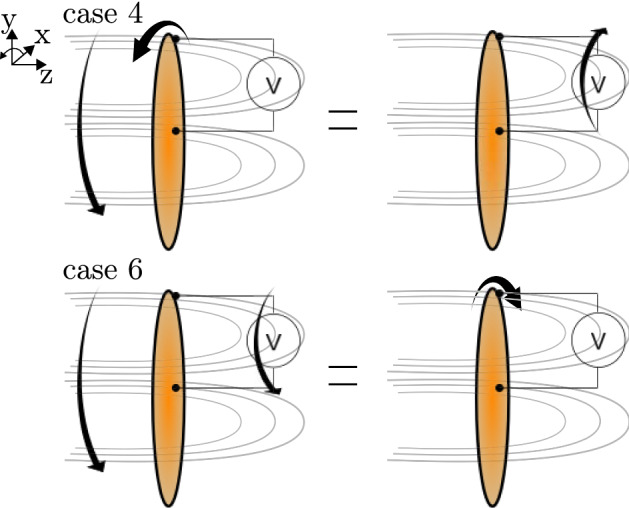
Relative motion of the magnetic field (which is assumed as co-rotating) compared to the circuit.

These results, especially case 5 and the corresponding intensity measurements clearly show that the closing wire, respectively the measurement circuit, has a definite influence on the phenomenon and the full circuit needs to be considered when describing and analysing the problem. This avoids the creation of paradoxes and helps to clarify the physical causes of the phenomenon. It also confirms that the rotation of the magnet is not as significant to the problem as previous research has ascribed and only the relative motion between disc and closing wire/measurement device determines the appearance of an EMF. In cases 2 and 5 we can clearly locate the seat of induction in the part of the circuit that is moving, as in case 2 the disc gets polarised and in case 5 it is the closing wire. However, if we analyse cases 4 and 6 it is not possible any more to tell in which part of the circuit the induction is seated. If we assume the field to be stationary and analyse case 4 in CCW rotation and NP amplifier setting, we find that the disc will polarise and the electrons will flow into the inverting input. If we instead assume the field to be moving, from the perspective of the closing wire that is being cut by the field lines rotating CCW, the relative motion between the two is the same as if the closing wire was rotating in a stationary field in CW direction. Consequently, the closing wire will polarise in such a way that the electrons can flow into the inverting input, resulting in an observed voltage. This relative equality has been sketched in Fig. 7, where the same relative relationship between moving field and disc has been shown for case 6 in CCW rotation.

The same argument can not only be made for case 6 in CCW rotation (field and closing wire spinning CCW is the same as the disc spinning CW in a stationary field), but the inverse relationships are found when the field is considered rotating in all tests of CW direction. This means that we are not able to distinguish between the field co-rotating or remaining stationary from these results, as the outcome of both assumptions is the same and we cannot isolate the seat of induction in cases 4 and 6. At this point, the question about the motion of the field cannot be conclusively answered, both co-rotating field lines and stationary field lines appear as valid alternatives and it remains unclear if the motion of the field (around the symmetry axis) can be detected at all.

It seems to be precisely this ambiguity of the field’s state of motion that is responsible for a lasting debate in the literature and both arguments for and against the field’s movements have been presented. In favour of the field remaining stationary, the one-piece Faraday generator has been researched by Crooks et al.^25^ and interpreted as the field remaining stationary and the magnet cutting its own field lines upon rotation. Also Cramp et al.^20^ argued against the field’s movements on the basis that the field lines would move faster than the speed of light at infinity, therefore it must remain stationary. However, since there are other situations where the field clearly must follow the magnets movement, such as rectilinear motion or a horseshoe magnet or quadrupole magnet, it raises the question as to why rotation about the symmetry axis should pose an exception to that behaviour? What would be the physical reason responsible for this peculiar consideration? If, for example, we spin the bar magnet differently so that it does not rotate around its symmetry axis but its radial axis instead (so that the poles chase each other in succession) there would be no doubt that the field lines follow the position of the poles. In this regard, it might be feasible to investigate further setups with a one-piece Faraday generator as they could hold evidence concerning the field’s movement or allow further conclusions to be drawn about the seat of induction. For example, experiments where the measurement is in the far field or where a non-contact detection method is employed, such as an electric field measurement arising from the separation of charges on the surface of the conducting magnet when the one-piece generator is spinning on its own. Such an endeavour would of course not be trivial, as field meters utilise movement of capacitive elements, and as soon as a conductor moves in the magnetic field there is likely to be undesired effects obscuring the measurement of the radial electric field of the Faraday generator. However, if measurements of this kind are successful it could provide compelling insight regarding the movement of the field lines or even the physical reality of the field.

It is fascinating that the ambiguity of the physical field model sparks such debate due to arising paradoxes, although perhaps the better question to ask might be: “Is the field physically real or not?” Because if it is not a real entity, then it also obviously can neither move, co-rotate nor remain stationary and hence paradoxes are avoided. As fields cannot be measured independently of forces, no stand-alone direct proof exists that confirms the physical reality of the field. However, we know for sure that the force is real that acts between the charge carriers. There exists an approach in the literature to explain the phenomenon considering the motion of the conduction electrons and the conservation of energy instead of the field alone^62^. With such an approach the field does not necessarily need to be considered as a real physical entity, but rather as a mathematical tool indicating a vectorial map of possible interactions. Further, Slepian made the argument that only the knowledge of a local vector field with direction and magnitude is relevant to describe observable phenomena and field lines or ‘lines of force’ do not have to be continuous, individual or closed curves^71^. As was reviewed by Miller^72^ ‘lines of force’ are a tool for visualisation, but they are not necessarily a ‘fruitful approach’ and their sources must be considered, respectively ‘whether lines of force have physical characteristics’. This can also help in explaining results when other cases of (unipolar) induction are regarded where magnet and conductor are in relative motion, such as the Barnett experiment^11,16–18^. It was commented by O’Rahilly^73^ [Vol. 2, p. 603], that saying the field is stationary only means that the vectorial field $\mathbf {B}$ ‘remains the same at every point of space round a rotating symmetrical magnet’ and that stationary lines of force are ‘merely an out-of-date invention adopted for those who are supposed not to be able to grasp the mathematical idea of a vector field.’ In this sense, O’Rahilly is making the same argument as Miller, that the lines of force can be a misleading approach, distracting from the actual cause of induction and the field.

From the perspective of the Weber force, the phenomenon can also be explained readily without paradoxes and without conceptual problems. The microscopic circulating charges (i.e., Ampèrian currents) in the magnet give rise to permanent magnetism and thus a magnetic force, which is then felt by the parts of the circuit (consisting of disc, contacts, closing wire and measurement device) that move. The EMF can then manifest whenever there is relative motion between the disc and the closing wire, as previously predicted by Assis and Thober^69^, as summarised in Table 1. It is clear that, since Weber predicts the seat of the EMF to be in the part of the circuit that actually moves, this agrees with the experimental results. Weber’s original force formulation pre-dates Maxwell’s equations and has received significant attention from Assis^63,69,74–77^ along with other recent applications^78–89^. Since the field as a mediator is conceptually avoided in the direct-action theory, there is no associated paradox concerning the field motion. Furthermore, if the field is not real, then the field approach and direct-action approach are indeed very similar. Moreover, it has been shown that Weber electrodynamics can be construed in a way that is consistent with the general concept of fields and that Maxwell’s equations can be obtained therefrom^63,90–94^. Especially Kühn^94^ has strongly interpreted Weber’s force as a field theory and an approximation of Maxwell’s equations. Even though the principle of Ockham’s razor favours a direct-action approach in this sense, as it makes less assumptions and entities are not multiplied unnecessarily, since the field itself is not needed, only the charge motion, direct-action is rejected by most scientists due to the existence of electromagnetic waves (e.g., radio waves). While Weber’s theory is not without limitations^95–99^, it does present an elegant way to resolve apparent paradoxes connected to unipolar induction. As the Weber force is closely related to Ampère’s force it should also be noted that the applicability of Ampère’s force versus Grassmann’s force has been discussed in connection with homopolar generator and motor configurations^100–104^. On the one hand, some authors^100–102^ are of the opinion that Ampère’s force is necessary to explain torque generation in the Guala-Valvede generator and motor configurations^26,105^ as well as Kennard’s, Bartlett’s and Barnett’s experiments. On the other hand, the contrary opinion has been put forward that all of these experiments can be explained classically^103,104^, albeit a full re-analysis of the experiments with due regard to the influence of the closing wire might be necessary.

## Conclusion

We have devised a novel experimental arrangement for a Faraday generator that includes the relative motion of the measurement circuit itself. By using a sensitive amplifier circuit connected to an LED output that can be rotated, we are able to report new experimental evidence clearly demonstrating that the measurement circuit is critical and must be included in any discussion/analysis relating to this phenomenon. However, it is not possible to tell if the field co-rotates with the magnet or remains stationary, and it remains unclear if this question can be resolved at all. In practice, both hypotheses are valid alternative explanations to the phenomenon. Regardless of the field co-rotating with the magnet or not, unipolar induction is not a paradox if the closing wire is considered. Further questions about the reality of the field as a physical entity are discussed, because conceptual problems arise when considering any other motion of a magnet where the field must follow the magnet’s movement. If, however, the field is not real, consequentially it cannot move with the magnet in the first place, and it is rather the motion of charges (i.e., the electron dynamics) that is responsible for observable phenomena. In this way associated paradoxes can be resolved. Congruent to this line of enquiry, an explanation for unipolar induction that avoids conceptual paradoxes and correctly predicts the experimental results, can be obtained by considering the direct interaction forces between charges within the system.

## Supplementary Information

Supplementary Figures.

## Data Availability

The data that support the findings of this study are available from the corresponding author upon reasonable request.
