# Supplementary material for: Resolving the paradox of unipolar induction: new experimental evidence on the influence of the test circuit
Source: Sci Rep. 2022 Oct 6;12:16791. doi: 10.1038/s41598-022-21155-x (PMC9537145; doi:10.1038/s41598-022-21155-x)
Supplement: Supplementary file 1 — Supplementary Figures. [file 41598_2022_21155_MOESM1_ESM.pdf]

Supplemental Information

Resolving the Paradox of Unipolar Induction: New Experimental Evidence on the Influence of the Test Circuit

Christof Baumgärtel and Simon Maher *

Department of Electrical Engineering and Electronics, University of Liverpool, Liverpool, UK.

*Address correspondence to:
S. Maher at Department of Electrical Engineering and Electronics, University of Liverpool, Brownlow Hill, Liverpool, L69 3GJ, UK. E-mail: s.maher@liverpool.ac.uk

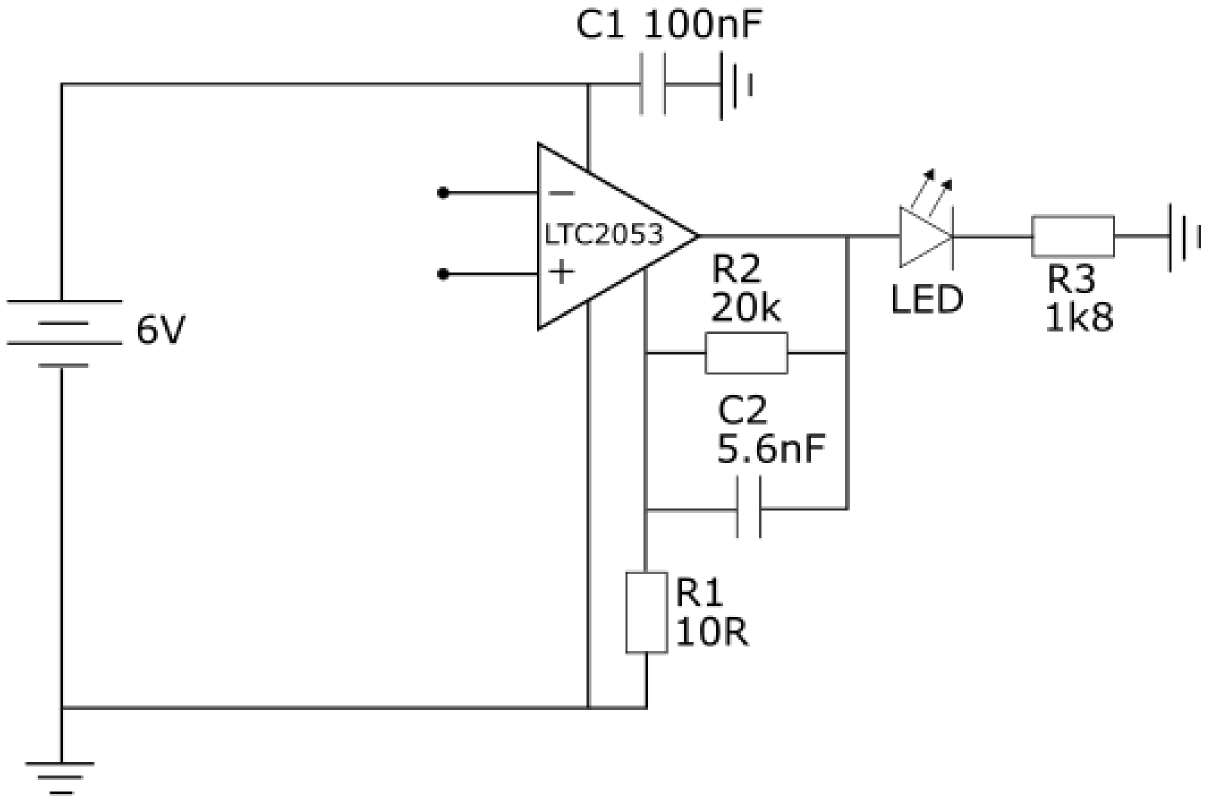

Figure S1: Circuit diagram of the detector circuit with amplifier and LED.

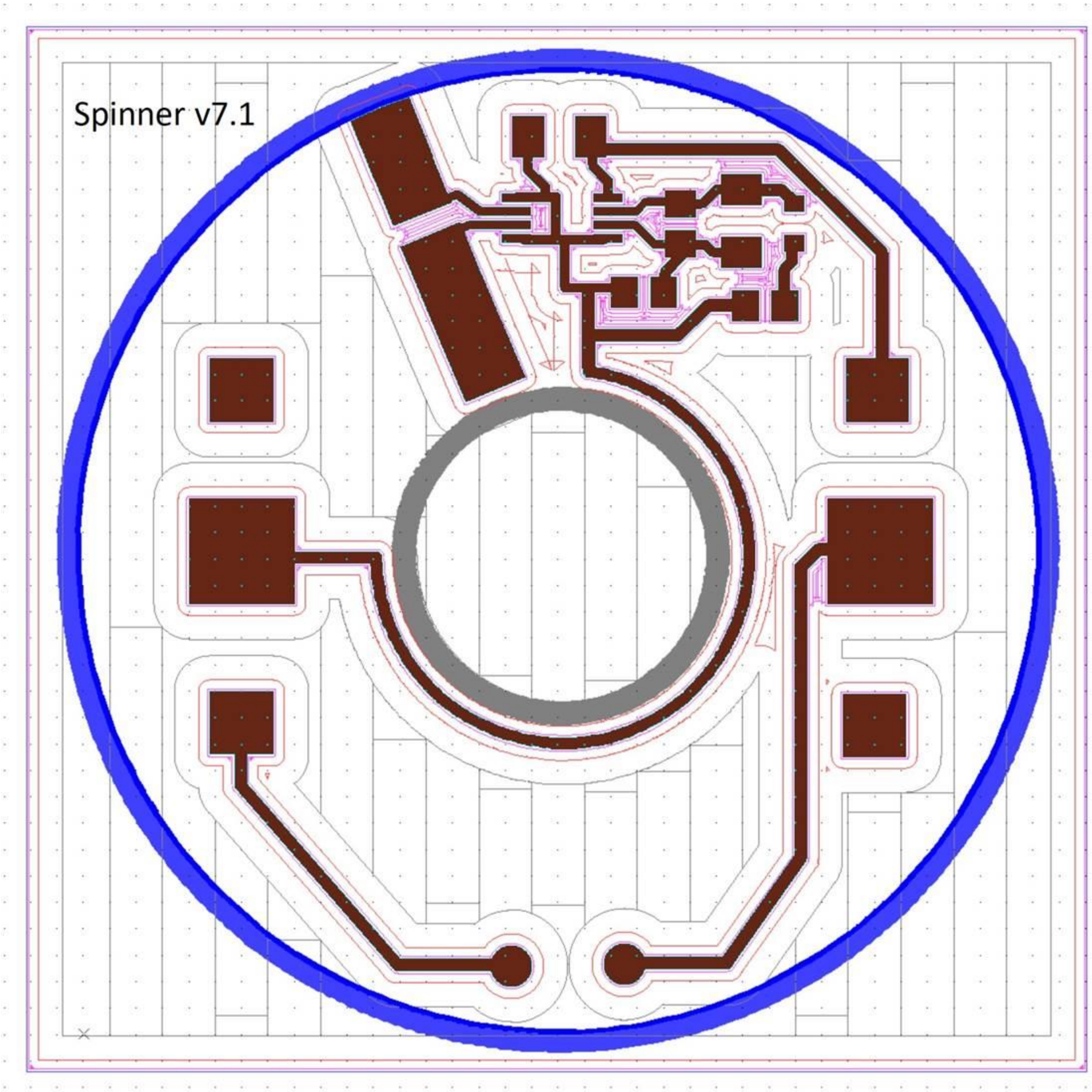

Figure S2: PCB layout.
